# Patient-Centered Diabetes Care Through an Ethical Lens: Associations with Patient Satisfaction and Patient-Reported Outcomes in Saudi Primary Healthcare

**DOI:** 10.3390/healthcare14183098

**Published:** 2026-09-20

**Authors:** Fahad Alhazmi

**Affiliations:** Department of Health Services and Hospitals Administration, Faculty of Economics and Administration, King Abdulaziz University, Jeddah 21589, Saudi Arabia; fahad.n.alhazmi@gmail.com or fnalhazmi@kau.edu.sa

**Keywords:** diabetes, patient-centered care, patient satisfaction, patient-reported outcome measures, patient-reported experience measures, primary care

## Abstract

**Background**: Patient-centered care (PCC) is central to diabetes management; unfortunately, evidence concerning its relationship with patient-reported outcome measures (PROM) in primary healthcare facilities in Jeddah is limited. This study examined the association between validated PCC scores and patient-reported outcomes and explored how PCC domains could be interpreted through four ethical principles. **Methods**: A cross-sectional survey was conducted on 594 adults with diabetes recruited from 47 primary care facilities between July and August 2023. The primary exposure was the validated 36-item, eight-domain PCC total score, while the primary outcome was a study-specific core PROM composite. Satisfaction and patient-reported experience measure (PREM) scores were measured as secondary measures. Statistical analysis was completed through multivariable ordinary least-squares regression with HC3 heteroscedasticity-robust standard errors adjusted for sociodemographic and health characteristics, while the four author-defined ethical groupings were examined exploratorily. **Results**: Among 594 adults with diabetes who completed the study, the mean PCC was 76.91 (SD 17.01), core PROM was 63.51 (SD 16.85), satisfaction was 81.41 (SD 19.30), and PREM was 77.27 (SD 20.45). PCC correlated with core PROM (*r* = 0.437), satisfaction (*r* = 0.251), and PREM (*r* = 0.239). In the primary adjusted model (*n* = 591), PCC remained positively associated with core PROM (*B* = 0.433, 95% CI [0.359, 0.507], *p* < 0.001). Female sex and severe self-rated health were associated with lower PROM scores, whereas older age and type 2 diabetes were associated with higher scores. The model explained 27.0% of PROM variance. **Conclusions**: Higher PCC was independently associated with better PREM. The ethical mapping provides a potentially useful interpretive framework but requires independent psychometric validation.

## 1. Introduction

Diabetes is a chronic medical condition characterized by consistently elevated blood glucose levels [1]. The International Diabetes Federation (IDF) lists diabetes as a key contributor to the global burden of disease with a prevalence of 529 million people and a cost of $966 billion in annual costs as of 2021 [1]. However, this burden has been rising at unprecedented rates since the early 1990s and is projected to hit 1.31 billion cases and more than $1.05 trillion by 2050 [1,2].

The pooled prevalence of diabetes in the Middle East is more severe, with a range of 15% to 16.2%, which is 1.5 times the global average [1,3]. The Kingdom of Saudi Arabia (KSA) is among the most affected countries in the Middle Eastern region, with 16.4% of its population meeting the criteria for diabetes [4]. Local studies have also revealed significant disparities across administrative provinces in the prevalence of diabetes across KSA [5]. Precisely, Jeddah is recognized as among the most affected governorates, with a diabetes prevalence of 18.3% [5]. As such, there is a need for care models that address both glycemic control and patients’ experiences, functioning, autonomy, and quality of life [6].

Diabetes presents significant challenges to contemporary health systems. This disease is recognized as the eighth leading cause of death and a key cause of severe complications, hospitalizations, years of life lost, diminished quality of life, and disabilities [1,2]. Persons diagnosed with diabetes require long-term coordination of clinical care, medication use, self-management, lifestyle support, complication screening, and psychosocial attention [7]. The survival and quality of life among persons with diabetes are dependent on the quality of care delivered by primary healthcare systems and the ability of providers to deliver care that patients can understand, trust, and use in daily life. Both international diabetes guidelines emphasize patient-centered care (PCC) as a key attribute of high-quality care for persons with diabetes. For instance, the Saudi National Diabetes Center (SNDC) guidelines and current evidence from the literature suggest the need for person-centered communication, shared decision-making, social context, and individualized treatment goals [7,8,9]. These agencies recognize patient-centeredness as a critical element that ensures the design and delivery of feasible, acceptable, and meaningful treatment plans [7,8,9].

PCC has a clear ethical dimension. This principle emphasizes respect for patient preferences and understandable information to ensure practical expressions of autonomy [10]. Additionally, providers must make efforts to promote beneficence and nonmaleficence by reducing discomfort, supporting coping, coordinating services, and responding to dynamic emotional needs. Further, PCC integrates justice through equitable access to care and continuity of care, particularly for underserved populations [11]. These ethical commitments are consistent with established accounts of PCC as an approach that is grounded in communication, values, and relationships rather than in isolated service transactions [10]. Scholars describe PCC as a multidimensional construct involving autonomy support, collaboration, communication, education, emotional support, and family or peer involvement [9]. The principle of PCC supports patient-reported outcome measures (PROMs) that explain how patients experience the burden of diabetes, treatment, daily functioning, worries, and support [12]. Studies show that PCC is generally associated with better patient satisfaction, improved communication, and better health outcomes, with varying effect sizes and causal pathways [10,13].

PCC can be understood through both quality of care and ethical perspectives [9]. Respect for autonomy is reflected in the presentation of understandable information, elicitation of preferences, and shared decision-making during diabetes management [9,10]. Diabetes care aligns with the principles of beneficence and nonmaleficence through attention to physical comfort, emotional well-being, and supportive involvement of patients’ families or friends [11]. Further, justice concerns equitable access to services, while relational accountability is achieved through coordination and continuity across the care pathway [11]. Although these ethical concepts do not constitute a validated measurement structure, they provide an interpretive framework for examining PCC.

Diabetes care in Saudi Arabia is delivered within a system undergoing continuing reform. This system has shown growing interest in chronic-disease management, patient experience, and service responsiveness [7,8]. Local studies have highlighted the importance of treating each patient with diabetes as a whole person rather than focusing solely on the diagnosis and the integration of PCC in self-care education to improve diabetes control in family-practice settings [14,15]. However, gaps have been noted between policy aspirations and patients’ lived experience of PCC [15]. Additionally, patient and provider satisfaction with PCC across the Middle East is shaped by access, communication, continuity, and interpersonal dimensions of care [16].

Evidence from KSA suggests that patients with diabetes value participation, understandable communication, individualized education, and recognition of their lived experience [6]. However, these patients describe implementation gaps that can limit their involvement in decisions and continuity of care [15]. At the same time, quantitative evidence linking validated PCC assessments with patient-reported outcomes in Jeddah primary healthcare remains limited.

Unfortunately, little is known about whether ethical dimensions of PCC in diabetes care are empirically associated with PROMs in Saudi primary healthcare despite this growing literature supporting it across other regions. For instance, a patient may still lack the information, continuity, and support needed to manage diabetes despite being satisfied with a visit. The same patient may also report a positive experience with some components of diabetes care while still facing broader barriers to everyday functioning. Scholars emphasize the need to distinguish these outcomes/experiences since satisfaction, PROMs, and patient-reported experience measures (PREMs) tend to answer different evaluative questions [17,18,19,20]. Many diabetes service evaluations report satisfaction due to its familiarity and ease of interpretation. However, the broad nature of satisfaction when used in isolation makes it difficult to identify elements of care that require improvement. Scholars suggest that measuring PREMs helps determine whether care processes occurred from the patient’s perspective, while the incorporation of PROMs can help determine whether patients reported better functioning, confidence, worries, and support [18,19].

Incorporating PROMs and PREMs constructs together can thus help identify ethically meaningful features of PCC that are associated with patient-reported outcomes after taking satisfaction and experience into account. The purpose of this cross-sectional survey was to examine whether the ethically interpretable PCC domains are associated with core PROM scores, describe satisfaction and PREM scores, and examine their correlations with PCC and PROM based on data collected from adults with diabetes attending primary healthcare centers in Jeddah, KSA. An exploratory objective to map the eight PCC domains onto four author-defined ethical principles and describe the resulting patterns was also considered.

## 2. Materials and Methods

This study followed a multicenter cross-sectional survey design. The survey was conducted across 47 primary healthcare centers affiliated with general hospitals in Jeddah, KSA. The study was reported in accordance with the Strengthening the Reporting of Observational Studies in Epidemiology (STROBE) statement (Supplementary Materials), while the Consensus-Based Checklist for Reporting of Survey Studies (CROSS) was used as a supplementary reporting guide for designing and reporting the survey study [21,22].

### 2.1. Sampling and Recruitment

Data was collected from 47 primary care facilities. The researchers used a consecutive, facility-based sampling technique to ensure fairness and inclusivity across participating facilities. Eligible participants included adults (aged 18 years and older), diagnosed with diabetes, receiving care in participating primary healthcare centers during the study period, and willing and able to complete the questionnaire. Eligible patients were approached consecutively as they attended participating centers during the data collection period. Participants were invited to participate in the study from all participating primary care facilities during the collection period.

An a priori power analysis for multiple linear regression based on Cohen’s (f^2^) effect-size framework was used to calculate the sample size. The researchers used data from the General Authority for Statistics and assumed a small incremental effect (f^2^ = 0.02), one tested predictor, 10 covariates, 80% power, and a two-sided significance level of 0.05 to estimate the minimum sample size [23,24]. It was determined that approximately 395 participants or more were needed to complete the survey. The researchers approached a total of 809 eligible patients to participate in the study to increase the external validity of the findings. However, 215 patients did not complete participation due to appointment-related time constraints, withdrawal, or insufficient time to complete the interview.

### 2.2. Data Collection and Survey Measures

The researchers trained research assistants to aid in data collection. These assistants assessed whether participants met the inclusion criteria, explained the purpose and procedures of the study, obtained verbal informed consent from each participant, read the questionnaire items aloud, and recorded participants’ responses. To maintain confidentiality, participants’ names and other directly identifying information were not recorded on the questionnaires. Participants were identified using study records rather than personal identifiers, and research assistants were instructed to maintain the privacy and confidentiality of all information provided by participants.

PCC was measured using 36 items covering patient preferences, physical comfort, coordination of care, continuity and transition, emotional support, access to care, information and education, and family and friends. This structure is consistent with the 36-item Patient-Centered Primary Care instrument for assessing patient-centered care in primary-care settings [25,26,27]. Patient satisfaction was measured using four items, including access, continuity and transition, behaviors of staff, and diabetes clinic services. PREM and PROM items captured patients’ experience with provider support and treatment/devices. These measures also covered worries, capabilities, barriers, support from others, and how patients felt about care. Covariates included age group, sex, nationality, education, monthly income, body mass index (BMI), diabetes type, and current health state. Questionnaires were conducted in English or Arabic based on each participant’s preferences, with a qualified translator helping translate those completed in Arabic into English for consistency and ease of analysis. Each questionnaire was then transferred into Microsoft Excel as a distinct record to facilitate aggregation and statistical analysis.

### 2.3. Ethical-Domain Construction

**Figure 1:**
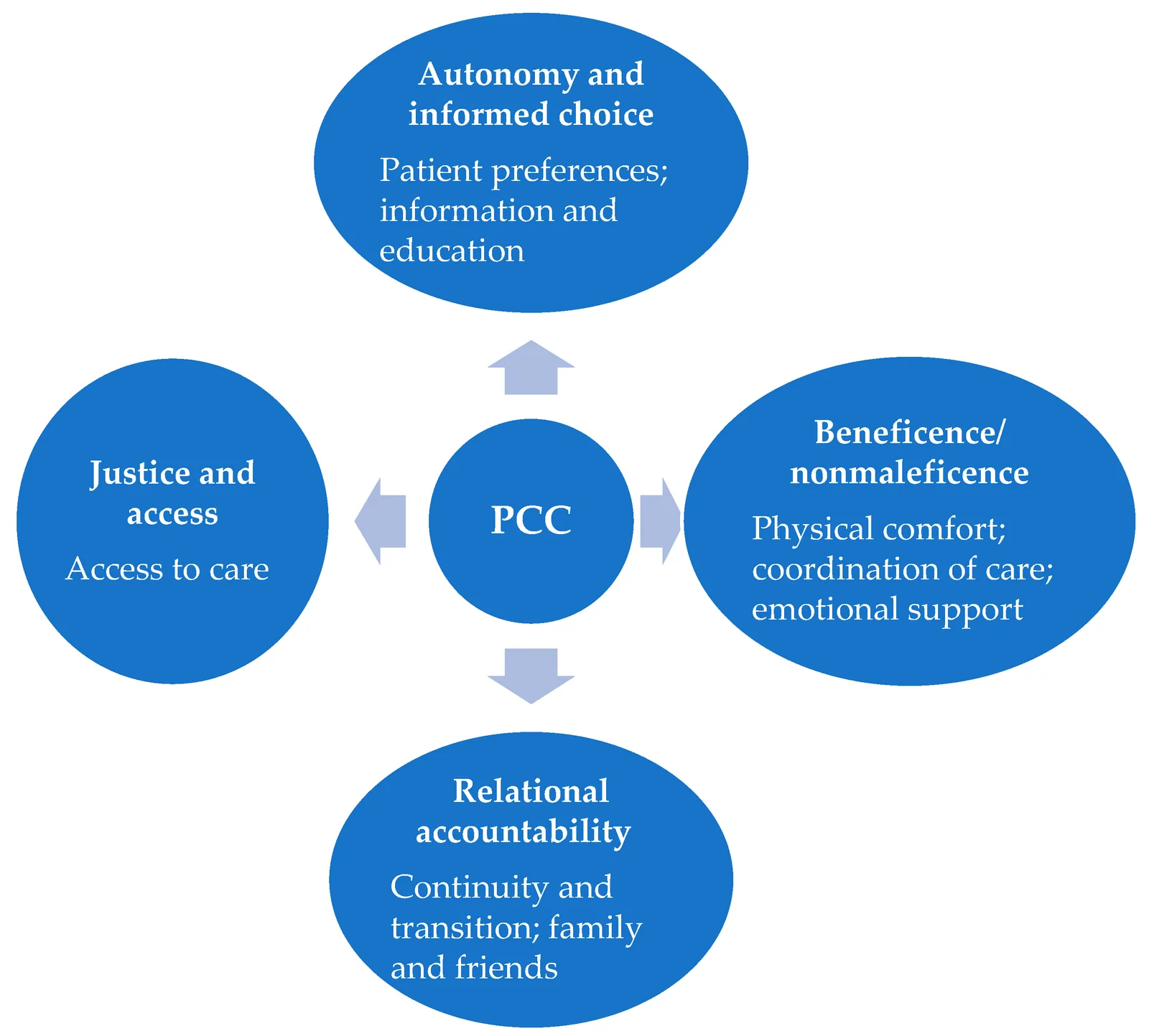
Modeling of PCC variables.

The primary exposure was the validated Arabic 36-item PCC instrument. This instrument comprises eight domains: patient preferences, information and education, physical comfort, emotional support, involvement of family and friends, access to care, coordination of care, and continuity and transition [25,26]. Notably, autonomy/informed choice combined patient preferences (PP) with information and education (IE), beneficence/nonmaleficence combined physical comfort (PC), coordination of care (CC), and emotional support (ES), relational accountability combined continuity and transition (CT) with family and friends (FF), and justice/access used the access-to-care (AC) domain (see Figure 1). This mapping provided a practical measurement bridge between bioethical principles and observable patient reports rather than replacing formal philosophical analysis. Items were transformed to a 0–100 scale, with higher scores indicating more favorable PCC. The total score was calculated as the mean of the four groupings’ scores according to the pre-specified scoring procedure. The four ethical groupings were created by the researchers for interpretive purposes and were not validated subscales. Each ethical score was calculated by averaging its constituent PCC domains, giving equal weight to each included domain. The internal consistency in the present sample was excellent (α = 0.961). The primary outcome was a study-specific core PROM composite comprising 16 consistently directed items addressing functioning and diabetes-related well-being.

### 2.4. Statistical Analysis

Descriptive statistics were generated to summarize respondent characteristics and scale distributions. Missing data were handled using available-case analysis for descriptive statistics and correlations and complete-case analysis for multivariable regression. No statistical imputation was performed. Three implausible BMI values were recoded as missing, and the primary adjusted regression included 591 participants with complete data for the outcome, exposure, and included covariates, which were excluded from the analysis. Cronbach’s alpha was also calculated to assess internal consistency, followed by Pearson correlations to describe relationships among the continuous PCC, satisfaction, PREM, and PROM 0–100 scores. The prespecified primary analysis examined the association between the validated eight-domain PCC total score and the core PROM score using ordinary least-squares regression with HC3 heteroscedasticity-robust standard errors. Covariates were selected a priori based on their plausible relationships with PCC or patient-reported outcomes. These covariates included age, sex, nationality, education, income, BMI, diabetes type, and severe self-rated health. The results were presented as unstandardized regression coefficients (B), standardized coefficients (β), HC3-robust standard errors, 95% CI, and two-sided *p*-values. Model performance was summarized using R^2^ and adjusted R^2^. Multicollinearity was then assessed using variance inflation factors and tolerance values, and residual plots were visually examined to evaluate model assumptions. The associations involving satisfaction and PREM and analyses of the four ethical PCC groupings were treated as exploratory. The exploratory subgroup comparison used independent-samples *t* tests or one-way analysis of variance (ANOVA), while statistical significance was defined as *p* < 0.05. The results were interpreted cautiously and based on effect sizes, CIs, and consistency rather than just statistical significance since multiple testing adjustments were not applied to the exploratory analyses. Finally, a complete-case analysis was used for each analysis, with reporting including applicable sample sizes. The analysis was performed using IBM’s Statistical Package for the Social Sciences, which is recognized as one of the most powerful analytical tools for health research [26].

### 2.5. Ethical Considerations

This study was approved by the Saudi Arabia Ministry of Health’s Central Institutional Review Board (IRB) at the Saudi Arabia Ministry of Health (log number: A01569) and the Ethics Review Committee at the Faculty of Health Medicine & Life Sciences at Maastricht University (Approval number: FHML-REC/2023/045). Additionally, verbal informed consent was obtained from all participants before data collection. Confidentiality was maintained throughout the study by avoiding the collection of participants’ names and other directly identifying information on the questionnaires. Research assistants were instructed to conduct the questionnaire administration privately and to protect the confidentiality of participants’ responses. Data were recorded using study records rather than personal identifiers and were analyzed and reported in aggregate form to minimize participant identifiability.

## 3. Results

### 3.1. Sample Characteristics

A total of 594 surveys were completed during the study, representing a response rate of 73.4%. The sample constituted 301 females (50.67%), predominantly Saudi nationals (85.35%), and mostly respondents with type 2 diabetes, mostly in stable current health status (93.4%), and mostly overweight or obese. However, BMI data was missing for 3% of participants. Table 1 below summarizes the sample’s demographics.

### 3.2. Correlation Among Main Measures

**Figure 2:**
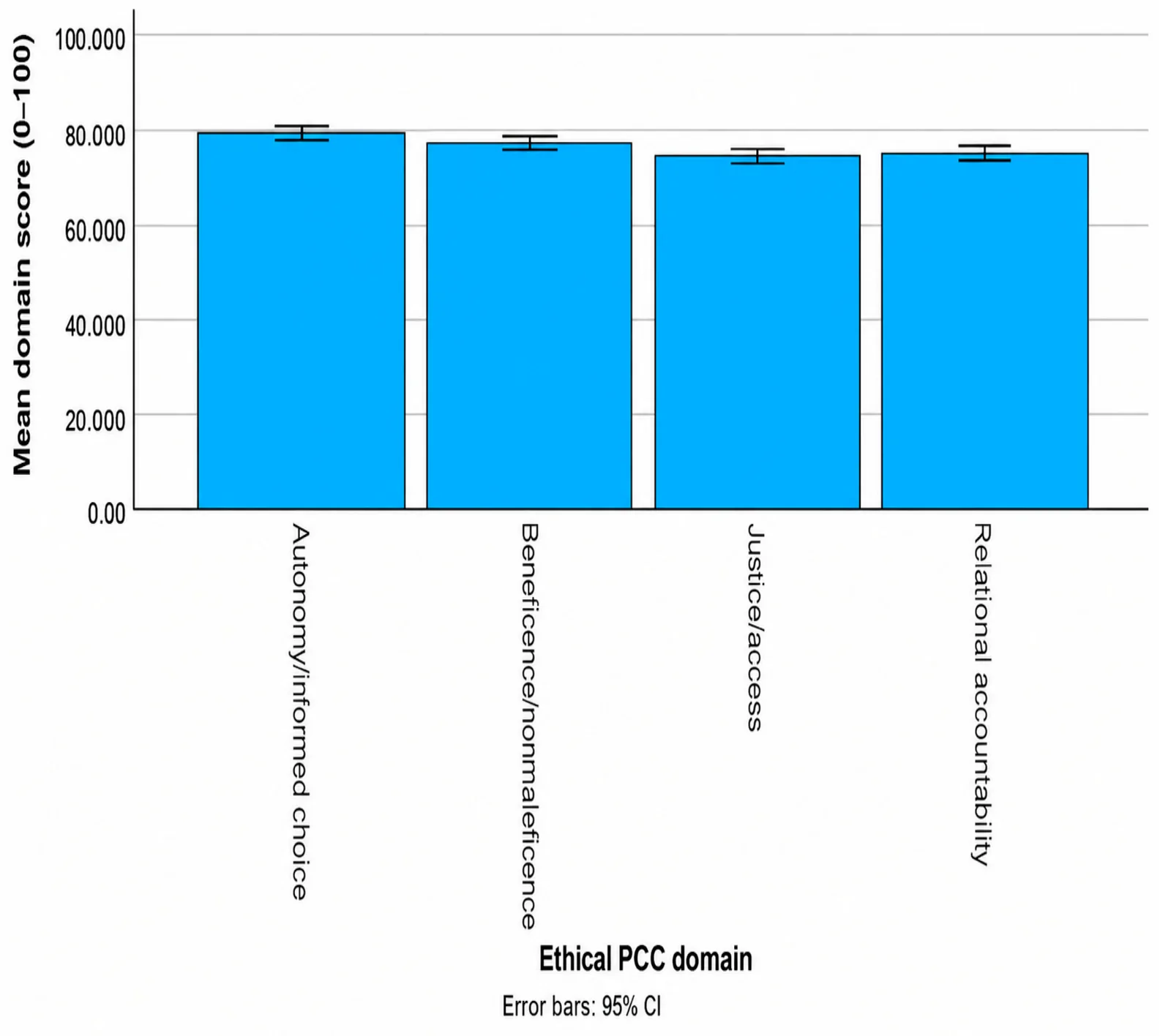
Ethical PCCs with 95% CI.

Mean validated PCC was 76.91 (SD 17.01), satisfaction was 81.41 (SD 19.30), total PREM was 77.27 (SD 20.45), and core PROM was 63.51 (SD 16.85). Internal consistency was excellent for PCC (α = 0.961) and acceptable for the core PROM (α = 0.843) and satisfaction scale (α = 0.825). As illustrated in Figure 2 below, autonomy and informed choice had the highest mean score (79.39), followed by beneficence/nonmaleficence (77.38), relational accountability (75.01), and justice and access (74.32) among ethical PCC domains scores 0–100.

**Figure 3:**
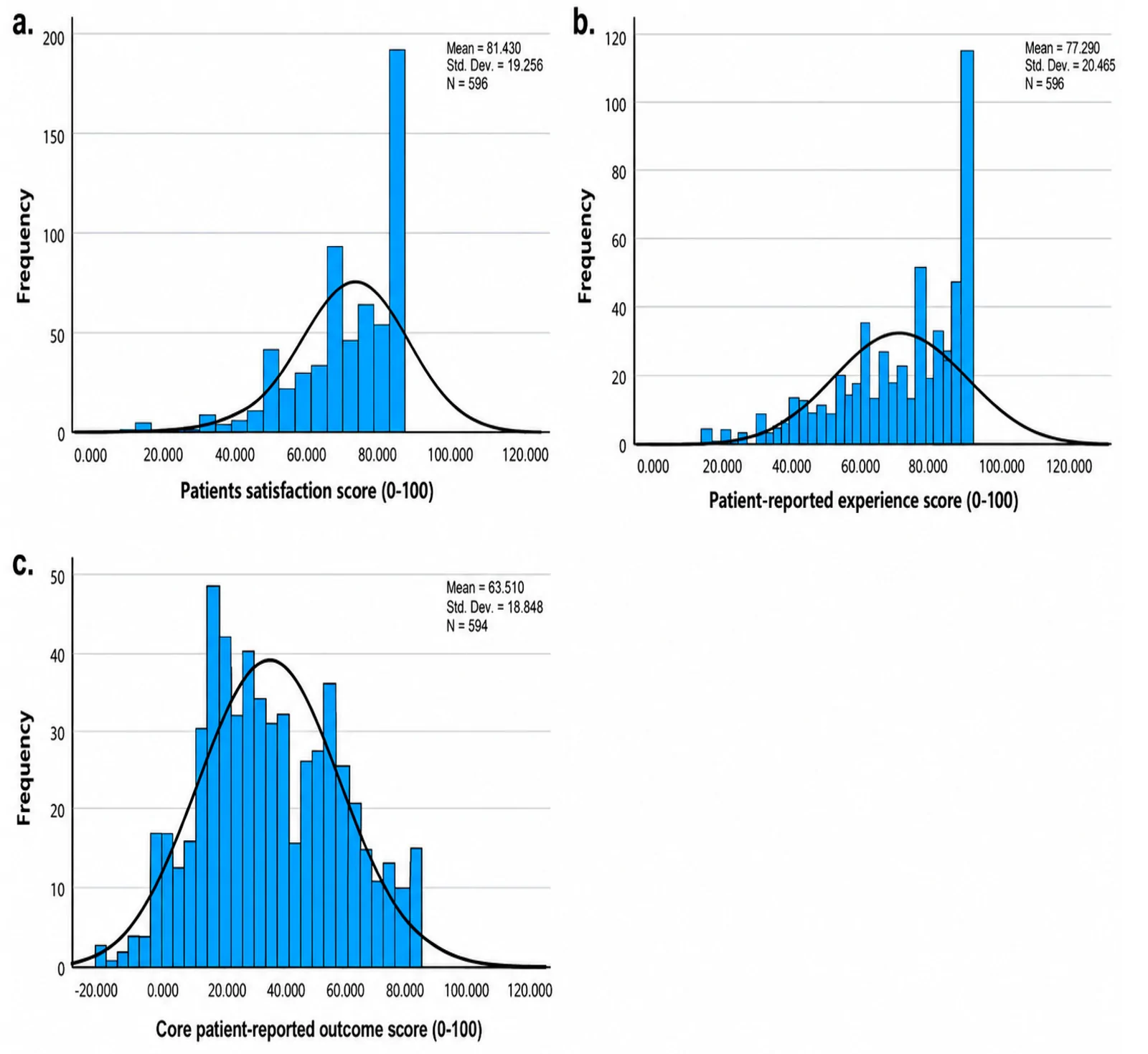
(**a**–**c**) Histograms showing score distributions.

Figure 3 below presents three histograms outlining various score distributions. Panels 3a, 3b, and 3c present patient satisfaction, PREM total, and core PROM scores. Score distributions demonstrated ceiling clustering for patient satisfaction and PREM, while the core PROM score was more broadly distributed.

### 3.3. Correlations Among PCC and Patient-Reported Measures

Pearson correlations showed a moderate positive association between overall ethical PCC and core PROM (r = 0.437, *p* < 0.001). There was a weak correlation between ethical PCC and satisfaction (r = 0.251, *p* < 0.001) and PREM total (r = 0.239, *p* < 0.001). Similarly, satisfaction and PREM were correlated with each other (r = 0.281), with each having a weak correlation with PROM at r = 0.128 and r = 0.134, respectively. These patterns supported the decision to examine PROM as a distinct endpoint rather than treating satisfaction or PREM as equivalent patient-reported outcomes. Further, all four ethical domains were positively correlated with PROM as follows: autonomy and informed choice (r = 0.427), beneficence/nonmaleficence (r = 0.409), justice and access (r = 0.395), and relational accountability (r = 0.348). The variability of these correlations suggested that the ethical domains captured related but non-interchangeable features of PCC in diabetes. These findings indicate that the measures were related but represented distinguishable aspects of patient-reported healthcare quality. Table 2 below presents a summary of these findings.

**Figure 4:**
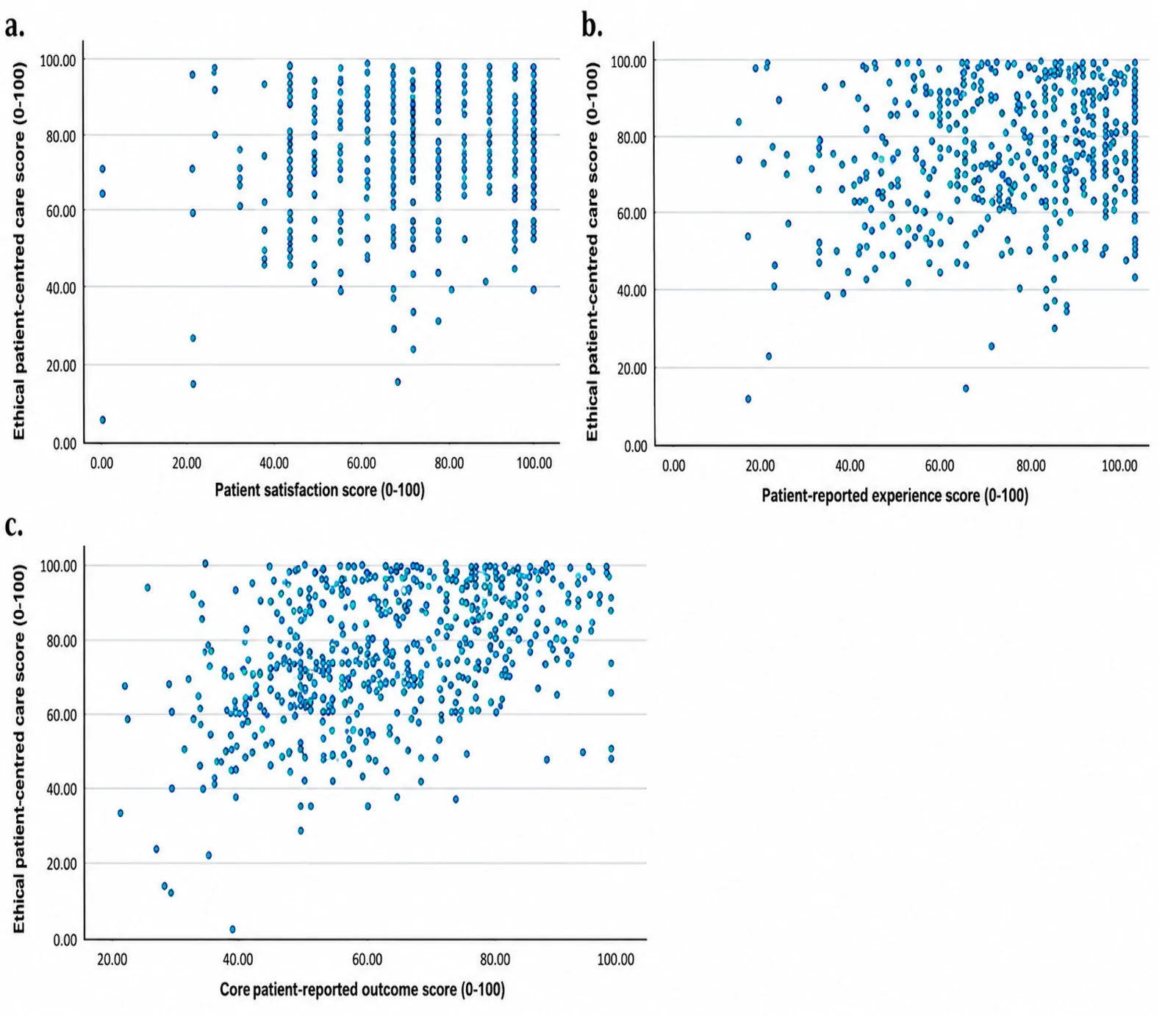
(**a**–**c**) Scatter plots representing ethical PCC vs. patient satisfaction.

Figure 4a–c below summarizes a visual analysis of the correlations. These scatterplots support the correlation findings, with the ethical PCC–PROM relationship appearing stronger than the relationships of ethical PCC with satisfaction or PREM. Panels 4a, 4b, and 4c show ethical PCC vs. patient satisfaction, PREM, and core PROM scores, respectively. The Pearson correlations for the three measures were r = 0.253, r = 0.239, and r = 0.442, respectively.

### 3.4. Bivariate Subgroup Findings

Several subgroup patterns were identified before adjustment. Notably, ethical PCC differed by age group (F = 5.72, *p* < 0.001, η^2^ = 0.028), education (F = 10.68, *p* < 0.001, η^2^ = 0.068), and income (F = 11.46, *p* < 0.001, η^2^ = 0.055). Core PROM differed by diabetes type (F = 7.46, *p* < 0.001, η^2^ = 0.025) and was substantially lower among persons with severe current health compared to those in stable health status (means 49.53 vs. 64.49; *p* < 0.001). These findings are summarized in Table 3 below.

### 3.5. Primary Adjusted Association Between PCC and PROM

In the HC3-adjusted primary model (*n* = 591), PCC remained independently associated with core PROM. Each one-point increase in PCC was associated with a 0.433-point increase in PROM (B = 0.433, 95% CI [0.359, 0.507], *p* < 0.001; standardized β = 0.437). The model explained 27.0% of PROM variance (R^2^ = 0.270), adjusted (R^2^ = 0.259). Female participants reported lower PROM scores than male participants (B = −4.278, 95% CI [−6.735, −1.821], *p* = 0.001), and severe self-rated health was associated with substantially lower PROM scores (B = −14.092, 95% CI [−18.763, −9.421], *p* < 0.001). Older age and type 2 diabetes were positively associated with PROM, whereas nationality, education, income, and BMI were not statistically significant. These findings are summarized in Table 4 below. B represents the unstandardized regression coefficient, and β represents the standardized coefficient. CIs and *p*-values were calculated using HC3 heteroscedasticity-robust standard errors. R^2^ = 0.270; adjusted R^2^ = 0.259.

**Figure 5:**
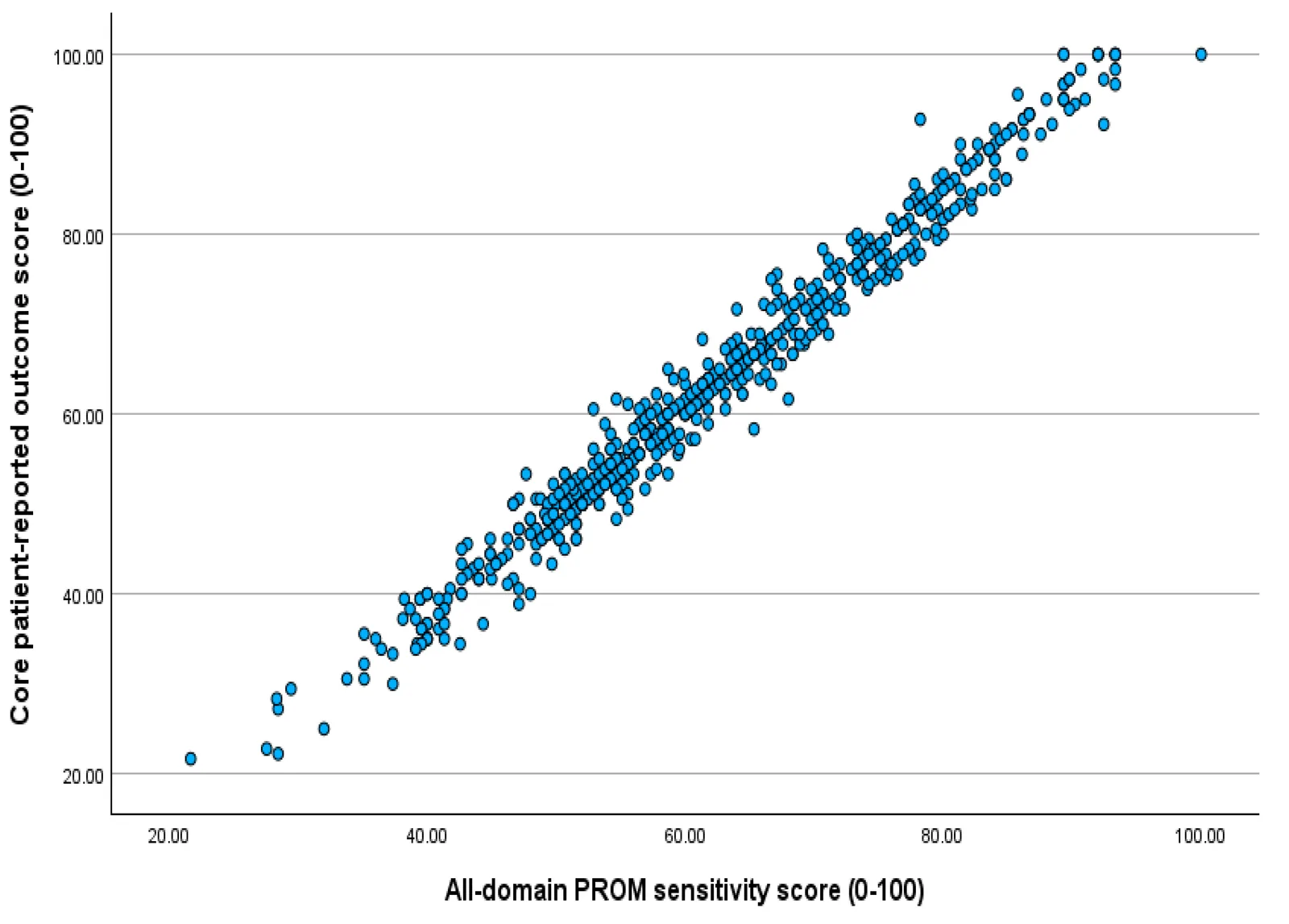
A scatter plot showing the sensitivity analysis of core PROM vs. all-domain PROM.

Figure 5 below shows the relationship between the core PROM and all-domain PROM sensitivity scores. The figure shows an almost perfect positive relationship between the core PROM and all-domain PROM scores (r = 0.989). The strong correlation is consistent with the shared items between the two measures.

## 4. Discussion

This cross-sectional study found that ethically organized PCC was independently associated with better PROMs among adults receiving diabetes care in PSA primary care facilities. Notably, the fully adjusted model revealed that each one-point increase in ethical PCC was associated with a 0.45-point increase in the core PROM score (see Table 4). The adjusted model explained almost a third (29.6%) of outcome variance. The robustness of the primary result was supported by the significant association that was maintained when the broader all-domain PROM score was used, which explained 32.3% of the variance. These findings are consistent with evidence from the literature that supports autonomy support, emotional support, collaboration, understandable education, and family involvement as key facilitators of patient activation and improve diabetes-related outcomes [9,12]. The findings add to qualitative evidence gathered in KSA, which showed the willingness of persons with diabetes to participate as partners of care rather than passive recipients of clinical decisions [15].

The study also revealed that patient satisfaction and PREM were positively correlated with ethical PCC, although the adjusted model did not find an independent association with PROM. This inconsistency is possible since PREM and PROM represented different aspects of healthcare quality. Notably, satisfaction and PROMs can be influenced by expectations, courtesy, and immediate impressions of a consultation [28,29]. While PROMs more directly assess how patients feel and function in everyday life, PREMs describe whether particular care processes occurred. Studies show a need to measure diabetes-specific outcomes like psychological well-being, treatment burden, self-management, and social functioning since they matter most to patients with diabetes [17]. As such, satisfaction, PREM, and PROM should be regarded as complementary rather than interchangeable indicators.

The study’s subgroup findings add an important equity dimension. Notably, ethical PCC ratings varied by age, education, and income, with a positive association emerging between severe current health and poorer PROM scores. However, income and education demonstrated small but inverse gradients with care ratings. However, the small effects in these findings should not be interpreted as definitive evidence that higher education and income lead to poorer perception of PCC. Scholars suggest that participants’ responses may be influenced by expectations, health literacy, and service-navigation experience beyond income and education variables [30]. Nevertheless, socioeconomic factors may have a significant impact on diabetes risk, access to resources, and disease management, which could shape the perceptions that patients develop towards PCC [31]. As such, more studies should be conducted to investigate education and income as mediators of satisfaction and PREM and PROM scores.

The above findings are clinically significant since they support the incorporation of ethical PCC indicators into routine diabetes quality assessment. Incorporating these indicators could enable primary healthcare facilities to monitor understandable information, shared decision-making, emotional support, access, continuity, and family involvement [9,10,12]. The findings from this study are consistent with evidence from past studies conducted in KSA that revealed that PCC self-care education can improve diabetes management and reduce mean HbA1c by up to 1.5% among patients with uncontrolled diabetes in just six months [14]. Although the current cross-sectional study cannot establish the causal relationship between ethical PCC and improved patient outcomes, its findings support prospective intervention studies.

This cross-sectional study has several strengths and limitations. Some of the strengths include its large multicenter sample, the incorporation of multiple patient-reported measures, the use of robust regression methods, sensitivity testing, and pathway and moderation analyses that raise the rigor of research. Unfortunately, the cross-sectional design reduces the rigor of research and prevents conclusions about temporality or causality, self-reported data raise the risk of bias, recruitment of participants from one city limits the generalizability of findings across the KSA population, and the absence of clinical variables like HbA1c limits the validity of findings [30]. Additionally, consecutive facility-based recruitment in one city limits population representativeness and generalizability beyond Jeddah. All principal measures were self-reported and collected during the same interview, creating potential social-desirability, interviewer, recall, and common-method biases. Within-center correlation could not be quantified using intraclass correlation coefficients or addressed using cluster-robust or multilevel models since facility identifiers were not retained. The unavailability of clinical information like HbA1c, diabetes duration, complications, treatment intensity, and comorbidity burden also results in potential residual confounding factors. Further, the core PROM was a study-specific analytic composite rather than the complete validated national diabetes register outcome score. Finally, the four ethical PCC groupings were author-defined interpretations of validated PCC domains and have not undergone independent construct validation. As such, ethical-domain findings should be considered exploratory and replicated using confirmatory psychometric methods.

## 5. Conclusions

This cross-sectional survey revealed that higher validated PCC scores were independently associated with better patient-reported outcomes among adults with diabetes receiving care at primary healthcare centers in Jeddah, KSA. Although satisfaction and PREM emerged as useful descriptive constructs, adjusted regression models revealed that these constructs did not statistically explain the ethical care-PROM association. These findings support monitoring communication, patient preferences, comfort levels, emotional support, family involvement, access to diabetes PCC, coordination of diabetes care, and continuity alongside clinical diabetes indicators. The exploratory ethical mapping offers a potentially useful interpretation of these dimensions of diabetes care. However, this framework should not be regarded as a validated four-domain instrument. As such, longitudinal and intervention studies incorporating clinical outcomes, probability-based recruitment, facility-level information, and independently validated ethical measures are needed to determine whether improvements in PCC can lead to better patient-reported outcomes.

## Figures and Tables

**Table 1:** Descriptive statistics.

Characteristic	Category	*n* (%)
Age	18–20	35 (5.9)
	21–40	121 (20.4)
	41–60	254 (42.8)
	61 or older	184 (31.0)
Gender	Female	301 (50.7)
	Male	293 (49.3)
Nationality	Saudi	507 (85.4)
	Non-Saudi	87 (14.6)
Education	Illiterate	99 (16.7)
	Secondary school or less	257 (43.3)
	Diploma	43 (7.2)
	Bachelor	157 (26.4)
	Postgraduate	38 (6.4)
Monthly income	5000 SAR or less	362 (60.9)
	5001–10,000 SAR	110 (18.5)
	10,001–15,000 SAR	62 (10.4)
	More than 15,000 SAR	60 (10.1)
Diabetes type	Type 1	169 (28.5)
	Type 2	406 (68.4)
	Gestational	19 (3.2)
Current health status	Stable	555 (93.4)
	Severe	39 (6.6)
BMI category	Underweight	17 (2.9)
	Healthy weight	141 (23.7)
	Overweight	175 (29.5)
	Obesity	258 (43.4)
	Missing	3 (0.5)

**Table 2:** A summary of Pearson correlations.

Correlations									
		Ethical PCC Score	Ethic Autonomy/Informed Choice	Ethic Beneficence/Nonmaleficence	Ethic Justice/Access	Ethic Relational/Accountability	Patient Satisfaction Score	Patient-Reported Experience Score	Core Patient-Reported Outcome Score
Ethical patient-centered care score	Pearson Correlation	1	0.900	0.903	0.839	0.920	0.253	0.239	0.442
	Sig. (2-tailed)		<0.001	<0.001	<0.001	<0.001	<0.001	<0.001	<0.001
	N	594	594	594	594	594	588	586	594
Ethic Autonomy/Informed Choice	Pearson Correlation	0.900	1	0.804	0.640	0.781	0.241	0.225	0.427
	Sig. (2-tailed)	<0.001		<0.001	<0.001	<0.001	<0.001	<0.001	<0.001
	N	594	594	594	594	594	588	586	594
Ethic Beneficence/Nonmaleficence	Pearson Correlation	0.903	0.804	1	0.625	0.810	0.228	0.215	0.409
	Sig. (2-tailed)	<0.001	<0.001		<0.001	<0.001	<0.001	<0.001	<0.001
	N	594	594	594	594	594	588	586	594
Ethic Justice Access	Pearson Correlation	0.839	0.640	0.625	1	0.686	0.212	0.186	0.395
	Sig. (2-tailed)	<0.001	<0.001	<0.001		<0.001	<0.001	<0.001	<0.001
	N	594	594	594	594	594	588	586	594
Ethic Relational Accountability	Pearson Correlation	0.920	0.781	0.810	0.686	1	0.223	0.222	0.348
	Sig. (2-tailed)	<0.001	<0.001	<0.001	<0.001		<0.001	<0.001	<0.001
	N	594	594	594	594	594	588	586	594
Patient satisfaction score	Pearson Correlation	0.253	0.241	0.228	0.212	0.223	1	0.281	0.128
	Sig. (2-tailed)	<0.001	<0.001	<0.001	<0.001	<0.001		<0.001	0.002
	N	588	588	588	588	588	588	583	588
Patient-reported experience score	Pearson Correlation	0.239	0.225	0.215	0.186	0.222	0.281	1	0.134
	Sig. (2-tailed)	<0.001	<0.001	<0.001	<0.001	<0.001	<0.001		0.001
	N	586	586	586	586	586	583	586	586
Core patient-reported outcome score	Pearson Correlation	0.442	0.427	0.409	0.395	0.348	0.128	0.134	1
	Sig. (2-tailed)	<0.001	<0.001	<0.001	<0.001	<0.001	0.002	0.001	
	N	594	594	594	594	594	588	586	594

**Table 3:** A summary of bivariate subgroup analyses.

Comparison	*n*	Test	Statistic	*p*	Effect	Value
Age > ethical PCC	594	One-way ANOVA	5.72	<0.001	eta squared	0.028
Education > ethical PCC	594	One-way ANOVA	10.68	<0.001	eta squared	0.068
Income > ethical PCC	594	One-way ANOVA	11.46	<0.001	eta squared	0.055
Diabetes type > PROM	594	One-way ANOVA	7.46	<0.001	eta squared	0.025
Current health > PROM	594	Welch *t*-test	−5.82	<0.001	Cohen d group2 minus group1	0.910
BMI category > PROM	591	One-way ANOVA	0.81	0.486	eta squared	0.004

**Table 4:** Primary regression table.

Predictor	B	Standardized β	Robust 95% CI	*p*
PCC total	0.433	0.437	0.359, 0.507	<0.001
Age category	1.636	0.083	0.050, 3.222	0.043
Female sex	−4.278	−0.127	−6.735, −1.821	0.001
Non-Saudi nationality	−2.155	−0.045	−5.571, 1.260	0.216
**Education**	−0.276	−0.020	−1.473, 0.920	0.650
**Income**	−0.687	−0.041	−2.193, 0.819	0.371
Body mass index	−0.129	−0.052	−0.302, 0.044	0.144
Type 2 diabetes	4.454	0.123	1.661, 7.248	0.002
Severe self-rated health	−14.092	−0.208	−18.763, −9.421	<0.001

## Data Availability

The raw data supporting the conclusions of this article will be made available by the author on request.

## Supplementary Materials

The following supporting information can be downloaded at: https://www.mdpi.com/article/10.3390/healthcare14183098/s1.

## Abbreviations

The following abbreviations are used in this manuscript:

KSA	The Kingdom of Saudi Arabia
PCC	Patient-centered care
SNDC	Saudi National Diabetes Center
PROM	Patient-reported outcome measures
PREMS	Patient-reported experience measures
PP	Patient preferences
IE	Information and education
PC	Physical comfort
CC	Coordination of care
CT	Continuity and transition
FF	Family and friends
AC	Access-to-care
ANOVA	Analysis of variance
IRB	Institutional Review Board
