# Supplementary material for: Patient-Centered Diabetes Care Through an Ethical Lens: Associations with Patient Satisfaction and Patient-Reported Outcomes in Saudi Primary Healthcare
Source: Healthcare (Basel). 2026 Sep 20;14(18):3098. doi: 10.3390/healthcare14183098 (PMC13606532; doi:10.3390/healthcare14183098)
Supplement: Supplementary file 1 [file healthcare-14-03098-s001.zip › healthcare-4494938-supplementary.pdf]

STROBE Checklist

	Item No	Recommendation	**Location where addressed in manuscript**
**Title and abstract**	1	(*a*) Indicate the study's design with a commonly used term in the title or the abstract	Title, p. 1: "A Cross-Sectional Study"; Abstract Methods, p. 1.
		(*b*) Provide in the abstract an informative and balanced summary of what was done and what was found	Structured abstract, p. 1.
Introduction			
Background/rationale	2	Explain the scientific background and rationale for the investigation being reported	Introduction, pp. 1–3.
Objectives	3	State specific objectives, including any prespecified hypotheses	Final paragraph of Introduction, p. 3; primary, secondary, and exploratory objectives identified.
Methods			
Study design	4	Present key elements of study design early in the paper	Section 2.1, pp. 3–4: 47 Jeddah primary healthcare centers; July–August 2023.
Setting	5	Describe the setting, locations, and relevant dates, including periods of recruitment, exposure, follow-up, and data collection	Section 2.2, p. 4: adult patients with diabetes, inclusion criteria, consecutive facility-based recruitment.
Participants	6	(*a*) Give the eligibility criteria, and the sources and methods of selection of participants	Sections 2.3–2.4, pp. 4–6: PCC exposure, core PROM outcome, satisfaction/PREM, covariates, exploratory ethical mapping.
Variables	7	Clearly define all outcomes, exposures, predictors, potential confounders, and effect modifiers. Give diagnostic criteria, if applicable	Sections2.2-2.3, pp. 4–6; Table 2, pp. 8-10
Data sources/ measurement	8*	For each variable of interest, give sources of data and details of methods of assessment (measurement). Describe comparability of assessment methods if there is more than one group	Sections 2.2–2.3, pp. 4–5: standardized training/administration, privacy, consecutive recruitment.
Bias	9	Describe any efforts to address potential sources of bias	Section 2.2, p. 4: Cohen f^2 calculation; approximately 395 required and 591 analyzed; limitations p. 13.
Study size	10	Explain how the study size was arrived at	Sections 2.2-2.3, pp. 5-6: 0–100 transformations, BMI use, categorical covariates; Table 1, pp. 6-7.
Quantitative variables	11	Explain how quantitative variables were handled in the analyses. If applicable, describe which groupings were chosen and why	Section 2.4, pp. 5-6: correlations, covariate adjustment, exploratory tests.
Statistical methods	12	(*a*) Describe all statistical methods, including those used to control for confounding	Section 2.4, pp. 5-6: Welch tests/ANOVA and effect sizes; subgroup analyses designated exploratory.
		(*b*) Describe any methods used to examine subgroups and interactions	Section 2.4, p. 5-6: no imputation; complete-case analysis; analysis-specific n reported.
		(*c*) Explain how missing data were addressed	Sections 2.2 and 2.4-2.5, pp. 4-6: independence assumption stated; facility identifiers unavailable, so clustering could not be modeled; limitation p. 13.
		(*d*) If applicable, describe analytical methods taking account of sampling strategy	Not applicable in revised primary analysis. The near-duplicate all-domain PROM sensitivity analysis was removed since r = .989.
		(*e*) Describe any sensitivity analyses	Abstract and Section 3.1, pp. 1 and 6-7: 809 approached; 594 completed; 591 in primary adjusted model.
Results	Results	Results	
Participants	13*	(a) Report numbers of individuals at each stage of study—eg numbers potentially eligible, examined for eligibility, confirmed eligible, included in the study, completing follow-up, and analyzed	Sections 2.1-2.2, pp. 3-4: time/appointment constraints and withdrawal.
		(b) Give reasons for non-participation at each stage	Numeric flow reported on pp. 4, and 7

	Item No	Recommendation	**Location where addressed in manuscript**
		(c) Consider use of a flow diagram	Section 3.1 and Table 1, pp. 7–8; scale descriptives in Section 3.2 and Table 1, pp. 6-7.
Descriptive data	14*	(a) Give characteristics of study participants (eg demographic, clinical, social) and information on exposures and potential confounders	Table 1, pp. 6-7; Table 2, pp. 9-10 (analysis-specific n); Table 3 note, p. 9.
		(b) Indicate number of participants with missing data for each variable of interest	Section 3.2 and Table 2, p. 8: PCC, satisfaction, PREM, and PROM means and SDs.
Outcome data	15*	Report numbers of outcome events or summary measures	Unadjusted correlations: Section 3.3/Table 3, pp. 8–9. Adjusted estimates and 95% CIs: Section 3.4/Table 4, pp. 11-12.
Main results	16	(*a*) Give unadjusted estimates and, if applicable, confounder-adjusted estimates and their precision (eg, 95% confidence interval). Make clear which confounders were adjusted for and why they were included	Table 1, pp. 6-7 age, income, and BMI categories. Regression coding/reference categories described in Table 4 note, p. 12.
		(*b*) Report category boundaries when continuous variables were categorized	N/A: outcome is continuous and results are reported as absolute PROM-point differences.
		(*c*) If relevant, consider translating estimates of relative risk into absolute risk for a meaningful time period	Sections 2.3 and 3.5 ethical mapping pp. 4-5, p. 11
Other analyses	17	Report other analyses done—eg analyses of subgroups and interactions, and sensitivity analyses	Sensitivity analysis for four author-defined ethical groupings on sections 2.3 and 3.5 pp. 4-5 and p. 11
Discussion	Discussion	Discussion	
Key results	18	Summarize key results with reference to study objectives	Discussion pp. 12-13
Limitations	19	Discuss limitations of the study, taking into account sources of potential bias or imprecision. Discuss both direction and magnitude of any potential bias	Discussion, p. 12-13: cross-sectional design, sampling, common-method/social-desirability bias, clustering, residual confounding, measurement limitations.
Interpretation	20	Give a cautious overall interpretation of results considering objectives, limitations, multiplicity of analyses, results from similar studies, and other relevant evidence	Discussion, pp. 12-13.
Generalizability	21	Discuss the generalizability (external validity) of the study results	Introduction, p. 3; Discussion limitations, p. 13: Jeddah-specific facility sample and limited generalizability.
Other information	Other information	Other information	Informed consent, data availability, and conflict of interest disclosed on p. 14
Funding	22	Give the source of funding and the role of the funders for the present study and, if applicable, for the original study on which the present article is based	Funding statement, p. 14: no external funding.
